# The role of different social contexts in shaping influenza transmission during the 2009 pandemic

**DOI:** 10.1038/srep07218

**Published:** 2014-11-27

**Authors:** Marco Ajelli, Piero Poletti, Alessia Melegaro, Stefano Merler

**Affiliations:** 1 Bruno Kessler Foundation, Trento, Italy; 2 Dondena Centre for Research on Social Dynamics, Bocconi University, Milan, Italy

## Abstract

Evaluating the relative importance of different social contexts in which infection transmission occurs is critical for identifying optimal intervention strategies. Nonetheless, an overall picture of influenza transmission in different social contexts has yet to emerge. Here we provide estimates of the fraction of infections generated in different social contexts during the 2009 H1N1 pandemic in Italy by making use of a highly detailed individual-based model accounting for time use data and parametrized on the basis of observed age-specific seroprevalence. We found that 41.6% (95%CI: 39–43.7%) of infections occurred in households, 26.7% (95%CI: 21–33.2) in schools, 3.3% (95%CI: 1.7–5%) in workplaces, and 28.4% (95%CI: 24.6–31.9%) in the general community. The above estimates strongly depend on the lower susceptibility to infection of individuals 19+ years old compared to younger ones, estimated to be 0.2 (95%CI 0.12–0.28). We also found that school closure over the weekends contributed to decrease the effective reproduction number of about 8% and significantly affected the pattern of transmission. These results highlight the pivotal role played by schools in the transmission of the 2009 H1N1 influenza. They may be relevant in the evaluation of intervention options and, hence, for informing policy decisions.

Despite influenza transmission has been extensively studied, little is known about the differential transmissibility of influenza viruses in different social settings, e.g., households, schools, and workplaces. A wide literature exists aimed at understanding and quantifying social contacts between individuals in different social settings based on different techniques, e.g. surveys on contact patterns^1^,^2^,^3^, analysis of socio-demographic data^4^, time-use data^5^,^6^, and radio-frequency identification sensor systems^7^,^8^,^9^. However, due to the difficulty of gathering reliable epidemiological data describing how infection is transmitted from one setting to another, these techniques have not been used to estimate the relative importance of different social contexts in the spread of influenza.

Adequate epidemiological data on influenza transmission are available for contacts between household members^10^,^11^,^12^,^13^,^14^,^15^,^16^,^17^ and, since the 2009 H1N1 influenza pandemic, between schoolmates^18^,^19^,^20^. Although these elements have been investigated individually in previous work through various modeling studies and statistical techniques, an overall picture has yet to emerge. Influenza transmission in different social contexts (including, for instance, workplaces and the general community) remains poorly understood; in fact, previous modeling studies^14^,^21^,^22^,^23^,^24^,^25^,^26^,^27^,^28^,^29^ have been mainly based on educated assumptions, rather than on empirical estimates, although evaluating the relative proportions of transmission in the different social contexts is of paramount importance for identifying the most optimal intervention strategy. Indeed, the uncertainty regarding the contribution of the various settings at different stages of the epidemic process clearly limits the ability to properly evaluate the efficacy of interventions such as closure of schools/workplaces, household quarantine, case isolation, and antiviral treatment. In this work we aim to fill this gap.

Human-to-human influenza transmission depends on i) frequency of contacts between individuals, ii) duration of the contact, and iii) intensity and type of contact – in terms of virus transmission interactions between children at school are substantially different from that of, for instance, adults in workplaces. A highly detailed individual-based model parametrized using realistic socio-demographic and time-use data is developed in this work and used to account directly for the first two components of influenza transmission (frequency and duration of contacts in different social settings, e.g. household, schools, workplaces, and the general community) and indirectly estimating the transmission rate given an adequate contact. To do this Bayesian statistical techniques are employed here to analyze serological data collected before and after the 2009 H1N1 influenza pandemic in Italy^30^,^31^. This analysis allows us to parametrize the model and to estimate the fractions of infections generated in different social settings. More in detail, Italian time-use data inform on individuals routine during the day and the time spent in different social contexts of 55,773 individuals. The analysis of time-use data enable, at any given time step of the simulation, and according to the day of the week, to dynamically associate individuals either to one specific location (e.g., their own household, their own school, etc.) or to the general community. Here contacts in the general community are defined as all contacts not occurring between household members, schoolmates, and work colleagues; so, for instance, the general community accounts for contacts occurring on public transportation, restaurants, shops, etc. Accounting for the time spent by individuals of different ages in different social contexts and employment types allows us to mimic the complex heterogeneous mixing of individuals within the population. Similar approaches have already been proposed, for instance in^5^,^6^,^21^,^32^ for studying airborne-transmitted diseases (like smallpox and influenza) and for deriving synthetic contact matrices by age. Such a high level of detail allows us to disentangle the contribution of the distinct social settings in the spread of the 2009 influenza pandemic. This information will be critical in deciding future control policies that will maximize the effectiveness of intervention strategies.

## Results

### Characterizing human behavior

**Figure 1:**
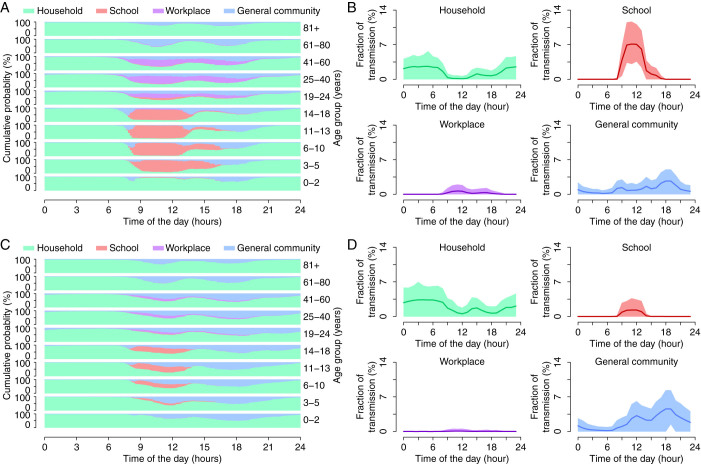
Daily time use and impact on disease transmission. (A) Cumulative probability of being in different social settings (household, school, work, general community) at each time of the day (with time step 10 minutes) during work days for different age groups. We considered only time-use data collected from September 1 to May 31 to exclude potential effects of summer vacations. This is coherent with the timing of the 2009 H1N1 pandemic in Italy, characterized by a single epidemic wave from September 2009 (with the reopening of schools after summer holidays) to January 2010. (B) Predicted average (lines) and 95%CI (colored areas) of the hourly percentage of daily transmission in different social contexts during work days. Results refer to infections occurring at day 48 of the simulated epidemics in a population of 100,000 individuals and with parameters as estimated for the 2009 influenza pandemic. (C) and (D) Same as (A) and (B), but for weekends. Differently from many other countries, most schools in Italy are open on Saturdays.

As can be derived from Fig. 1A, the individual routine during a work day (Monday to Friday) is very much dependent on the occupational status (e.g., student, worker, retired/unemployed). Indeed, during the morning, school-aged individuals spend their time at school mixing with peers of the same age, i.e. mixing is assortative by age. Similarly, a large fraction of adults spend their time at work whereby they tend to have contacts with colleagues. This also implies that during the morning, contacts in households and in the general community mainly involve retired/unemployed individuals (i.e., mainly the elderly). During the daytime, adults and children spend most of their time in the general community during lunchtime hours and before and after work/school time, including the time required for commuting from home to school/work and vice versa. The elderly spend a considerable amount of time in the general community (3.5 hours per day per individual aged 65+ years), with a peak of activity in the central part of the morning when students are generally attending classes and the presence of adults is marginal. During the late evening, and even more overnight, the mixing pattern is mainly characterized by contact between household members (about 95% of all contacts occurring between midnight and 6am is between household members).

Such a pattern of human activity is one of the main determinants of the infection process in different social settings. As clearly shown in Fig. 1B, according to model predictions, infections occur in different social contexts at different hours of the day – the overall transmission is also variable during the course of the day, see Supplementary Information – with peaks of transmission in schools during the morning, in the general community during the evening, and in households overnight. Simulations also show a peak of transmission in the general community early in the morning, ascribable to contacts in the time required to commute from home to school/workplace.

One aspect of influenza transmission not clearly analyzed yet is the role of weekends. Weekends could contribute to breaking the chain of transmission in schools and workplaces, because influenza generally has a short generation time – about the length of a weekend – thus influencing the overall pattern of spread. Therefore, the weekly calendar was expressly considered to account for the effect of weekends. The activity of individuals is regulated in such a way as to cyclically follow time-use data collected on workdays for five simulated days and then to follow time-use data collected on weekends for the next two simulated days. Specifically, we define the activity of individuals during a weekend day to be the activity reported in the time-use survey during Saturdays and Sundays, without distinguishing between them. Weekends are characterized by much more time spent in the general community (3.4 hours per day per individual during work days compared to 4.8 hours per day per individual on weekends) and much less at school (5 hours per day per student during school days compared to 1.7 hours per day per student on weekends) or work during the daytime (see Fig. 1C) resulting in a larger fraction of cases generated in the general community in the late morning and afternoon (see Fig. 1D).

Our analysis also highlights that, even if all individuals spend some time in the general community, mixing patterns in this setting are far from being homogeneous. In fact, different age groups spend their time in the general community at different times of the day, thus lowering the transmission probability of airborne diseases between different age groups.

### Age-specific seroprevalence

The epidemic transmission process is modeled according to a classic susceptible-latent-infectious-removed (SLIR) epidemiological model, describing virus transmission in the general community (R), with explicit transmission in households (H), schools (S), and workplaces (W). At any given time step, infectious individuals can infect only susceptible individuals who are sharing their same location at the same time (see Methods section and Supplementary Information). Specifically, three different disease transmission models characterized by an increasing level of complexity and realism of the social structures have been considered, namely:

- Model HR: only households and the general community are modeled and the latter accounts also for school and workplace contacts;
- Model HSR: households, schools, and the general community are modeled and the general community accounts also for workplace contacts;
- Model HSWR: households, schools, workplaces, and the general community are modeled.

**Figure 2:**
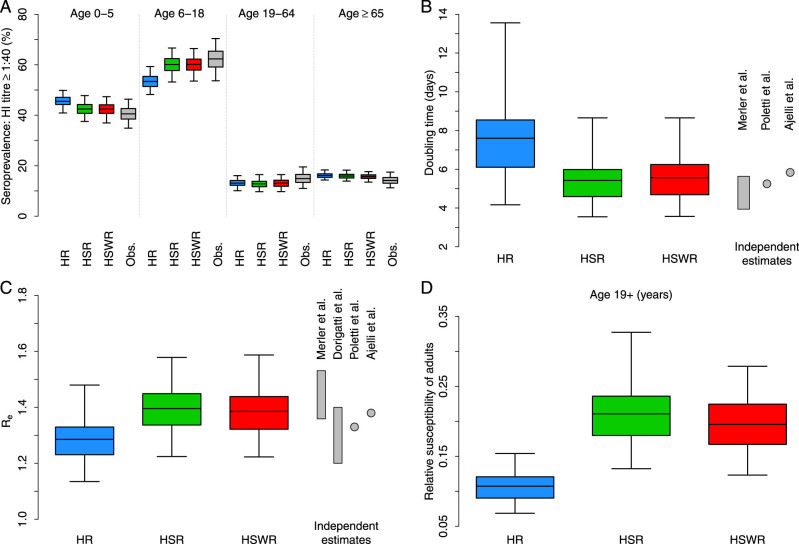
Model calibration and validation. (A) Distribution (mean, 50%CI and 95%CI as resulting from exact binomial tests) of the post-pandemic influenza seroprevalence by age group observed in the serological samples (grey; a sample is considered seropositive when HI titer is ≥ 40) and posterior distribution (mean, 50%CI and 95%CI) estimated with transmission models HR (blue), HSR (green) and HSWR (red). (B) Posterior distribution (mean, 50%CI and 95%CI) of the epidemic doubling time estimated with transmission models HR (blue), HSR (green) and HSWR (red). The grey bar refers to range estimated an independent study (Merler et al.^31^); grey points refer to the average values estimated in Poletti et al.^33^ and in Ajelli et al.^34^. (C) Posterior distribution (mean, 50%CI and 95%CI) of the effective reproduction number estimated with transmission models HR (blue), HSR (green) and HSWR (red). Grey bars refer to range estimated in independent studies (Merler et al.^31^ and Dorigatti et al.^37^); grey points refer to the average values estimated in Poletti et al.^33^ and in Ajelli et al.^34^. (D) Posterior distribution (mean, 50%CI and 95%CI) of the relative susceptibility to infection of adults (19+ year-old) with respect to younger individuals estimated with transmission models HR (blue), HSR (green) and HSWR (red).

We found that the two models explicitly considering transmission in schools (models HSR and HSWR) both perform significantly better than the simple structure model (model HR) in reproducing the observed post-pandemic age-specific seroprevalence (Fig. 2A). Model performances were evaluated by the Deviance Information Criterion (DIC; we recall that models with smaller DIC should be preferred). The DIC of the three considered models is 39.3 for model HR, 33.6 for model HSR, and 31.7 for model HSWR. Model HR results in overestimating the seroprevalence in pre-school children and underestimating seroprevalence in children and adolescents (see Fig. 2A). As for models HSR and HSWR, results show a significantly higher seroprevalence in school-aged children and adolescents (around 55–65%) – a high fraction of seropositive individuals (around 35–45%) is also estimated in pre-school children – compared to older age classes. These estimates compare well with observed data (see Fig. 2A). The fraction of H1N1 seropositive among elderly individuals does not significantly increase with respect to the pre-pandemic baseline (see Supplementary Information). All in all, the analysis suggests that 1) schools may have played a pivotal role in the transmission of influenza – this is why models HSR and HSWR, explicitly accounting for transmission in schools, outperform model HR – and 2) younger individuals could have been more susceptible, for either biological or behavioral reasons, to the disease than adults – this is why the inclusion of workplaces in the model does not result in significantly better estimates with respect to model HSR.

### Epidemic doubling time

The epidemic doubling time is the time required for the number of new daily cases to double their value, that is log(2)/*r* where *r* is the exponential growth rate of the incidence of new infections. The exponential growth rate of the simulated epidemics has been computed by fitting a linear model to the logarithm of the predicted daily incidence of new infections over a time windows of two weeks chosen in the initial phase of the epidemic, when the depletion of susceptibles is negligible and the incidence grows exponentially.

Estimates of the doubling time are 7.6 days (95% Credible Interval, CI: 4.3–13.6 days) for model HR, 5.4 days (95%CI: 3.6–8.7 days) for model HSR, and 5.6 days (95%CI: 3.6–8.7 days) for model HSWR. As for models accounting for explicit transmission in schools, although they are calibrated only on the basis of seroprevalence data, which does not include any direct information about the growth rate of the epidemic, they lead to estimates of the doubling time in satisfactory agreement with those reported in three independent studies^31^,^33^,^34^ (see Fig. 2B). Estimates provided by model HR are slightly larger than those reported in these studies (see Fig. 2B).

### Reproduction number

A key measure of the transmission potential of a disease is the reproduction number *R*_0_, which is defined as the average number of individuals infected by a typical infectious individual in a fully susceptible population. The effective reproduction number *R_e_* is used when a fraction of the population is already immune to the disease^35^. The procedure used for computing *R*_0_ is detailed in the Supplementary Information. As a fraction of the population, mainly concentrated in the elderly, was immune to the virus at the beginning of the 2009 pandemic, we provide estimates of *R_e_*.

We found *R_e_* = 1.29 (95%CI: 1.14–1.48) for model HR, 1.4 (95%CI: 1.23–1.58) for model HSR, and 1.39 (95%CI: 1.23–1.59) for model HSWR, in satisfactory agreement with independent estimates regarding the 2009 influenza pandemic in Italy, resulting from the analysis of different datasets and obtained by using different approaches^31^,^33^,^34^,^37^,^38^ (see Fig. 2C).

### Age-specific susceptibility to infection

One peculiarity of the 2009 influenza pandemic was an age-specific pattern of susceptibility to infection, as observed by studies focusing on the United States^15^, Mexico^41^, and European countries^31^,^38^. All these studies have highlighted remarkably larger relative susceptibility to infection in school-aged children and adolescents compared to adults and the elderly. Differential susceptibility to infection by age is accounted by assuming that individuals aged 19+ years are exposed to a lower force of infection with respect to younger individuals (see Methods for details).

In all tested models, we estimate a pattern characterized by lower susceptibility to infection in adults and the elderly compared to individuals 18 years of age or younger (see Fig. 2D), specifically 0.11 (95%CI 0.07–0.15) for model HR, 0.21 (95%CI 0.14–0.33) for model HSR, and 0.2 (95%CI 0.12–0.28) for model HSWR. Estimates provided by models HSR and HSWR (about 0.2 on average for all individuals 19 years of age or older) compare well with values reported in^31^.

These findings reveal the pivotal role played by schools in the transmission of the 2009 H1N1 influenza, acting as amplifiers of influenza transmission. In order to reproduce the observed profile of seroprevalence, model HR is forced to estimate a remarkably high (and hardly plausible) susceptibility to infection of school children and adolescents with respect to adults, in order to counterbalance the lack of contacts in schools between children and adolescents. In fact, as shown in^3^,^4^, these age groups are characterized by a higher number of contacts and a larger assortativity mainly due to school contact. Results reported so far allow us to exclude model HR from the rest of the analysis.

### Influenza transmission by setting

**Figure 3:**
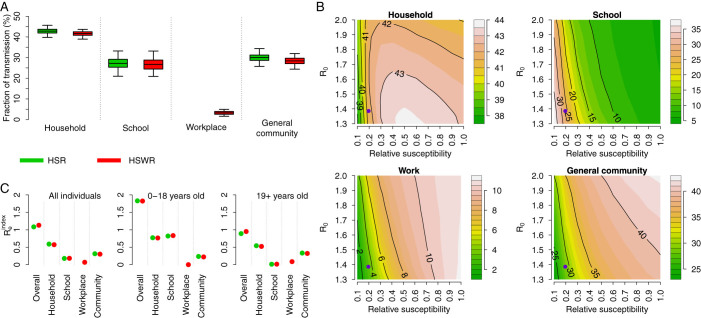
Influenza transmission in different social settings. (A) Posterior distribution (mean, 50%CI and 95%CI) of the proportion of transmission in households, schools, workplaces and in the general community estimated with transmission models HSR (green) and HSWR (red). (B) Proportion of transmission in different social settings as obtained by simulating transmission model HSWR and by varying *R*_0_ and susceptibility to infection of adults (19+ year-old) relative to younger individuals. Violet points represent central estimates of *R_e_* and susceptibility to infection of adults for the 2009 H1N1 pandemic in Italy. Results are obtained by averaging over 1,000 model simulations for each pair *R*_0_-relative susceptibility. Color scale on the right side of each panel. (C) 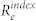, overall and in different settings, for all individuals (left subpanel), individuals aged 0–18 years (middle subpanel), and individuals aged 19+ years (right subpanel). Green points refer to model HSWR and red points to model HSR.

According to model HSR, the resulting fraction of transmission per setting is 42.8% (95%CI: 39.9–45.6%) in households, 27.2% (95%CI: 21.1–33.2%) in schools, and 30% (95%CI: 25.9–34.3%) in the general community (which also accounts for workplace infections); by assuming model HSWR the figure becomes 41.6% (95%CI: 39–43.7%) in households, 26.7% (95%CI: 21–33.2) in schools, 3.3% (95%CI: 1.7–5%) in workplaces, and 28.4% (95%CI: 24.6–31.9%) in the general community. Results are summarized in Fig. 3A.

Estimates obtained by assuming either model HSR or HSWR do not differ much; this is a consequence of the low level of transmission associated with contacts between work colleagues, as estimated by model HSWR. Such a low proportion of transmission within workplaces has already been found in^38^ and is possibly ascribable to the low susceptibility to infection of adults compared to that of younger individuals.

Lower values of within-household transmission (about 33%) have been estimated for the 1999–2000 influenza season in France (see Ferguson et al.^14^). Similar results have also been reported in^17^ for seasonal influenza (estimated range 22%–35%) and for pandemic influenza (estimated range 23%–37%) in Hong Kong. However, as hypothesized in^22^ and later confirmed in^42^, the fraction of transmission occurring in households strongly depends on the socio-demographic structure of the population where the virus spreads. The average age of the population and the household size are both factors critically affecting these estimates. Moreover, simulations show a high variability of estimates over time, especially in the initial phase of the epidemic when the number of cases is still low and the epidemic follows highly stochastic chains of infection. This should be cautiously taken into account when analyzing field data.

Estimates of the proportion of transmission in households are not very sensitive to *R*_0_ and adults susceptibility to infection compared to that of younger individuals, but the proportion of transmission in schools, in the general community, and to a lesser extent, in workplaces are (see Fig. 3B). We found that for each value of *R*_0_ the proportion of transmission in households is higher for intermediate values of relative susceptibility (about 0.3–0.5). Higher values of relative susceptibility result in much more transmission in the general community (more than 40% of cases generated in the general community) and much less transmission in schools (less than 10% of cases generated through school contacts). The opposite effect is observed when considering lower values of relative susceptibility, with less than 30% of cases generated in the general community and more than 35% of cases generated through school contacts.

Together with *R*_0_, another measure of the transmission potential of a disease is represented by *R^index^*, which is defined as the average number of individuals infected by the first infectious individual (the index case) in a fully susceptible population^22^,^36^. The effective 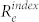 is used when a fraction of the population is already immune to the disease. The use of 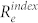 allows us to investigate the transmission potential by age groups. The procedure used for computing 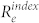 is detailed in the Supplementary Information.

As shown in Figure 3C, the estimated 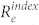 of the overall population is 1.13 by assuming model HSWR (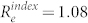 by assuming model HSR). Such a value is lower than the estimated effective reproduction number as the initial infective individual is randomly chosen and it cannot thus be considered a “typical” infector, as required in the definition of *R_e_* – the difference between the estimated *R_e_* and 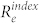 is in line with literature values^22^,^36^. Our results show that younger individuals (especially students) have a higher transmission potential than adults. Specifically, we found 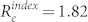 by assuming model HSWR (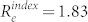 by assuming model HSR) for individuals aged 0–18 years, which is about twofold than that of individuals aged 19 years or more (specifically, 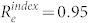 by assuming model HSWR and 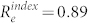 by assuming model HSR). This result is in agreement with the current knowledge on the 2009 influenza pandemic, suggesting that school age individuals have shown a higher transmission potential than adults (see for instance^39^,^40^). Moreover, we can also observe the relative contribution of the different settings to the overall 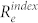. By looking at the entire population, we found that 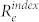 is 0.57 in households, 0.18 in schools, 0.07 in workplaces, and 0.3 in the general community according to model HSWR (0.59 in households, 0.18 in schools, and 0.31 in the general community according to model HSR). A part from obvious differences in the transmission potential at school, we found that 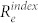 in household is 0.77 for individuals aged 0–18 years according to model HSWR (0.77 by assuming model HSR), while for individuals aged 19+ years it is 0.52 according to model HSWR (0.54 by assuming model HSR). This remarkable difference stems from the fact that younger individuals more likely live in larger households and with other young individuals^4^.

### The role of weekends

To assess the impact of weekends on the spread of the 2009 H1N1 pandemic we compare the above results to those obtained from a theoretical scenario where changes in individuals' habits during the weekend are not considered, i.e., weeks consist of seven work days. Results show that weekends (with more time spent in the general community and much less at school and work) are responsible for a reduction of *R_e_* of 6.7% on average according to model HSR (without weekends *R_e_* increases to 1.5, 95% CI 1.31–1.72), and a 8% reduction on average according to model HSWR (without weekends *R_e_* increases to 1.51, 95%CI 1.33–1.73). We also found that the fraction of cases in different settings is affected by weekends. According to model HSWR, weekends contribute to increase transmission in households of 3.5% (without modeling weekends the fraction of cases in households decreases to 40.2%, 95% CI 37.9–41.9), to decrease transmission in schools of 19.3% on average (without weekends the fraction of cases in schools increases to 33.1%, 95% CI 26.4–40), to decrease transmission in workplaces of 23.3% (without weekends the fraction of cases in workplaces increases to 4.3%, 95% CI 2.4–6.4), and to increase transmission in the general community of 26.8% on average (without weekends the fraction of cases in the general community decreases to 22.4%, 95% CI 19.5–25.3). Similar results were found with model HSR (see Supplementary Information).

While the estimated difference of transmission in households is not epidemiologically very relevant, results show that weekends may be responsible for a drop of about 19–25% of influenza transmission in schools. However, on the other side, a higher average proportion of transmission in the general community and, to a lesser extent, in households is also associated to weekends. This may be particularly relevant when considering school closure policies.

## Discussion

In this work we analyzed age-specific seroprevalence data collected in Italy pre and post the 2009 H1N1 influenza pandemic to identify the main routes of influenza transmission and to quantify their relative importance. Our results suggest that the two main routes of infection were household contacts (accounting for about 42% of all infections) and school contacts (accounting for about 27% of all infections). Only a negligible fraction of infection has been associated with within-workplace transmission (about 3%) while about 28% of all infections have been due to contacts occurring in other social contexts (e.g., public transportation system, leisure places, shops, restaurants, etc.). These figures, which are quite stable under different modeling assumptions, must be considered specific to the 2009 H1N1 pandemic. In fact, remarkably low levels of susceptibility to infection are associated with adults and the elderly compared to that of younger individuals, and it is not clear whether such a pattern is specific to the 2009 pandemic or a common signature of influenza pandemics. We estimated adults and the elderly to be about 5 times less susceptible to infection than children and adolescents. This value of relative susceptibility to infection, however, may depend on factors both biological (e.g., related to the strain of the virus and the host immune response) and behavioral (e.g., interactions between children at school are substantially different for chance of virus transmission than that of, for instance, adults at work). However, at this time, it is not possible to quantify the relative contributions of these factors in determining the overall pattern of susceptibility to infection. Different values of *R*_0_ and, even more importantly, of relative susceptibility, give rise to completely different figures – transmission in schools may vary from 5% to 35% and transmission in the general community may vary from 25% to 45%. We also found that the transmission potential of younger individuals (aged 18 years at most) was about two times higher than that of adults. Moreover, our analysis highlights that weekends are responsible for a decrease of the effective reproduction number of about 8%. Moreover, we found different figures of transmission by setting during weekends, with a drop of transmission in schools, a higher proportion in the general community and in households. These findings may inform policies to optimize containment/mitigation measures when facing a new influenza pandemic.

## Methods

### Modeling population's demographic, social, and behavioral characteristics

We simulate a population of 100,000 individuals (roughly the average size of an Italian municipality). The model is informed with detailed socio-demographic data on the Italian population as described in^4^. Here we also consider that individuals can move among different locations during the course of the day on the basis of time-use data for the Italian population, stratified by age and employment, work days and weekends, at 10-minute time resolution. Details are provided in Supplementary Information.

### Disease transmission model

Influenza transmission is modeled according to the classic SLIR scheme: (S) susceptible, individuals who can acquire the infection; (L) latent, individuals who are infected but not able to infect yet; (I) infectious, individuals who are infected and able to infect; and (R) removed, individuals who are immune to the disease, for instance because they recovered from infection.

One of the most striking features emerged from the analysis of the 2009 pandemic has been a higher susceptibility to infection of children/adolescents with respect to adults^15^,^31^,^38^,^41^. On the other hand, differences in infectiousness by age have never been reported in the literature and this hypothesis has even been ruled out^15^. Therefore, according to the literature, we assume age-specific susceptibility to infection, but not age-specific infectiousness. We stress, however, that this does not necessarily imply that all individuals have the same transmission potential – what remains constant is the transmission probability given an adequate contact. In fact, for instance, students would have a higher transmission potential than the elderly, as they tend to have a large pull of contacts (e.g. at school), most of which are individuals with high susceptibility to infection. With regard to susceptibility to infection, the population is divided into two susceptibility age classes: children and adolescents (individuals aged 0–18) and adults (19+ year-old individuals).

As each individual at any time step of the simulation is located in a specific location, we assume that susceptible individuals can get infected only through contact with infectious individuals who are sharing the same place at the same time. Specifically, at any time step *t* of the simulation, any susceptible individual *i* has a probability 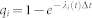 of being infected, where *λ_i_*(*t*) is the instantaneous risk of infection and Δ*t* is the length of the time step of the simulation. We assume homogeneous mixing between all individuals who are co-located in the same setting at the same time and thus the risk of infection can be computed at any time step of the simulation as: 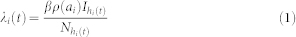
where

- *h_i_*(*t*) identifies the place where individual *i* is located at time *t*. *h_i_* can be the household of individual *i*, its school (if any), its workplace (if any), or the general community;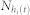 is the number of individuals co-located in place *h_i_* at time *t*. Thus, for instance, if *h_i_*(*t*) is a household, 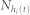 can be at most the number of household members;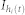 is the number of infectious individuals co-located in place *h_i_* at time *t*;
- *a_i_* is the age of individual *i*;
- *ρ* is the age-dependent susceptibility to infection of individuals. We assume that *ρ*(*a_i_*) = 1 if *a_i_* ≤ 18 to avoid over-parametrization and 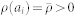 if *a_i_* > 18.
- *β* is the (setting independent) influenza transmission rate.

At each time step of the simulation, latent individuals enter the infectious phase with probability *ω*Δ*t*, where 1/*ω* is the average length of the latent period, which is assumed to be 1.5 days. Similarly, infectious individuals recover with probability *γ*Δ*t*, where 1/*γ* is the average length of the infectious period, which is assumed to be 1.2 days. This leads to a generation time of 2.7 days, in agreement with estimates given in the literature (see for instance^15^,^20^,^38^). Recovered individuals are assumed to have acquired full immunity to the circulating pandemic virus. Moreover, to account for the presence of immune individuals in the population before the pandemic, we randomly assign individuals to be in the removed class on the basis of the observed pre-pandemic age-specific seroprevalence rates^30^,^31^.

### Model calibration

The model has two free parameters: the influenza transmission rate *β* and the susceptibility to infection 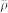 of adults (19+ years of age) compared to younger individuals (0–18 years of age). Posterior distributions of the free parameters were explored by Markov Chain Monte Carlo (MCMC) sampling applied to the likelihood of post-pandemic serological data reported in^31^. Specifically, by assuming that for each considered age group the number of positive samples is binomial *B*(*n*, *p*), the likelihood is defined as: 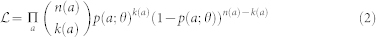
where index *a* runs over the age groups considered in the serological surveys (i.e., 0–5, 6–18, 19–64 and 65+ year-old individuals), *n*(*a*) is the number of samples tested, *k*(*a*) is the number of positive samples and *p*(*a*; *θ*) is the seroprevalence for age group *a* as resulting from model simulations with parameter set 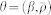.

## Author Contributions

S.M. and A.M. conceived the research, M.A. performed the computational work, M.A., P.P., A.M. and S.M. contributed to the interpretation of results and the writing of the manuscript.

## Supplementary Material

Supplementary Information Supplementary methods and additional results.
