# Supplementary material for: The role of different social contexts in shaping influenza transmission during the 2009 pandemic
Source: Sci Rep. 2014 Nov 27;4:7218. doi: 10.1038/srep07218 (PMC4245519; doi:10.1038/srep07218)
Supplement: Supplementary Information — Supplementary methods and additional results. [file srep07218-s1.pdf]

The role of different social contexts in shaping influenza transmission during the 2009 pandemic

Marco Ajelli[1], Piero Poletti[1,2], Alessia Melegaro[2] & Stefano Merler[1]

[1] Bruno Kessler Foundation, Trento, Italy
[2] Dondena Centre for Research on Social Dynamics, Bocconi University, Milan, Italy

Supplementary Information

Contents

1 Materials and Methods **2**
1.1 Socio-demographic model 2
1.2 Dynamic allocation of individuals in different settings 4
1.3 Calibration of the disease transmission model 7
1.4 Serological data 9
1.5 Computation of reproduction numbers 9
1.6 Computation of R^{index} and R_e^{index} 9

2 Additional results **12**
2.1 Robustness of results 12
2.2 Estimated influenza transmission rate 12
2.3 Within-day transmission dynamics 12
2.4 Influenza transmission by setting 13
2.5 The role of weekend 13

Bibliography **14**

1 Materials and Methods

The dynamic infection process is simulated through a discrete-time individual-based SLIR model that explicitly accounts for transmission occurring in households, schools, workplaces and in the general community. The model aims at reproducing the actual mixing of individuals in a population. The explicit modeling of households, schools and workplaces allows us to reproduce preferential contact patterns between individuals driven by their membership to specific clusters of individuals, e.g members of the same household or school mates. The resulting mixing by age is strongly assortative within schools and reflects generational age gaps within households, as already shown in [1, 2, 3]. What is novel here is that contacts between individuals occur only when they are sharing the same environment at the same time. The dynamic process is driven by time-use data collected for the Italian population at 10 minutes time resolution [4]. We found that individuals with different age and/or employment spend a different amount of time and at different time of the day in different social settings. This widely affects the mixing of individuals in the different social contexts.

1.1 Socio-demographic model

The simulated population of agents, each one corresponding to an individual of the actual population, is generated according to available descriptive statistics (e.g. age structure of the population, distribution of household size and composition, school attendance rates by age, employment rates by age, etc.) of the Italian population provided by the Italian Institute of Statistics and by the Statistical Office of the European Commission. All details about the algorithm used for simulating the socio-demographic structure of the population are available in [1, 2, 3].

Briefly, we use a heuristic model matching marginal distributions of household age by size, age of household members by size (and thus the age structure of the total population), and maintaining realistic generational age gaps between household members. The procedure used to build a household is the following. First, we determine household size by sampling from the distribution of household size. Then, we assign an age a_h to the household head, by sampling from the distribution of age for the specific household size, under the constraint that $a_h \geq 18$ years. For households having two, three or four members, we determine if there is a single adult or a couple, according to the corresponding probability for households of the assigned size. For the sake of simplicity, we do not consider households with more than two adults, e.g. a couple with children and an aggregate member. All households with more than four members are assumed to be composed by a couple with children, since type "couple with children" represents more than 95% of the total number of households. The age of the other household members is assigned by sampling from the age distribution for the specific household size, taking into account the following constraints:

(a) the age of the (possible) spouse, a_s, satisfies $\max\{a_h - 15, 18\} \leq a_s \leq \min\{100, a_h + 15\}$;

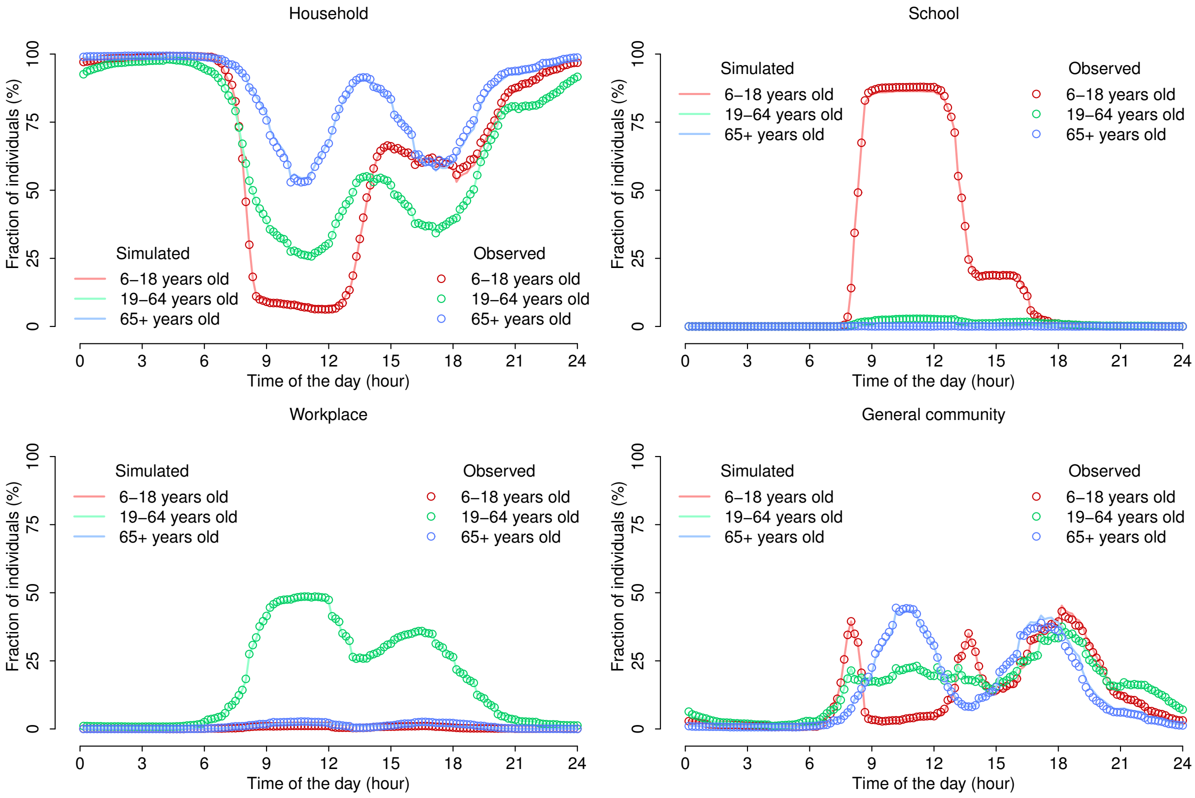

Figure S1: Simulated and observed (in the time-use survey) fraction of individuals in different social settings at different times of a work day.

(b) the age of (possible) children, a_c, in a household with a single adult, satisfies $\max\{0, a_h - 40\} \leq a_c \leq a_h - 20$;

(c) the age of (possible) children, a_c, in a household with two adults, satisfies $\max\{0, a_m\} \leq a_c \leq \min\{a_h, a_s\} - 20$, where $a_m = \max\{a_h, a_s\} - 40$.

The observed distribution of household size, age structures by household size, age structure of the population are accurately reproduced by the model, thus supporting our choice of neglecting non-private households or compositions such as families with aggregated members. The comparison between simulated and observed data is shown in [3].

As regards schools and workplaces, an occupation is assigned to each individual that either goes to school (as a student or teacher/school employee) or workplace, or remains at home (for instance as a retired or family worker) on the basis of age–specifc data on the employment rates. Every student is assigned to the correct school level, namely pre-primary (day-care centers, kindergartens), primary, lower secondary, and upper secondary schools, and higher education (post-secondary training, university, doctoral programs). We build in the model a certain number of schools (of the appropriate level) and workplaces such that all active individuals can be assigned to the appopriate school level or workplace.

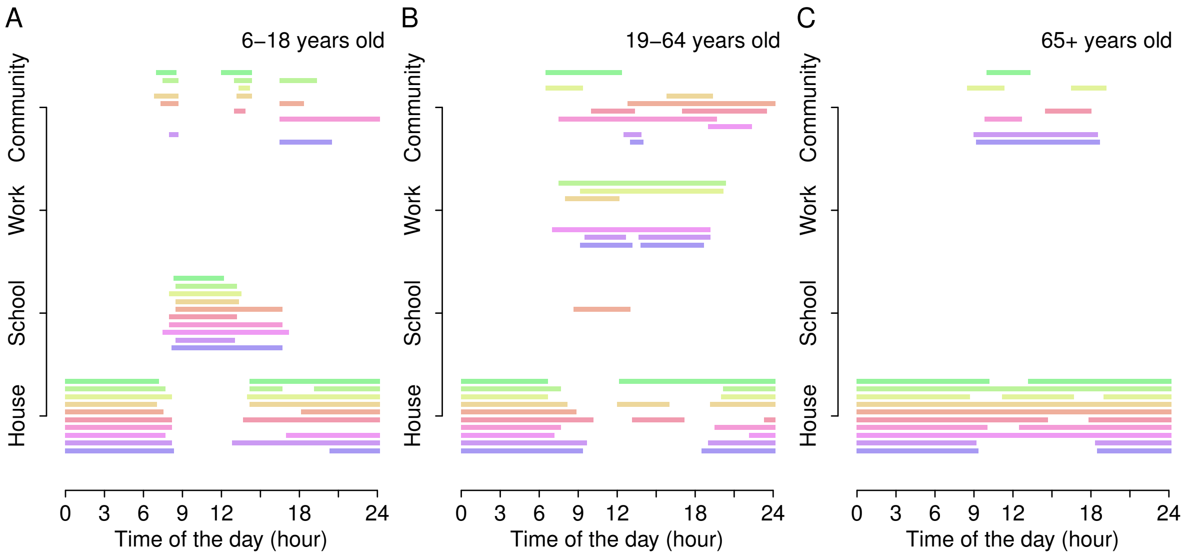

Figure S2: **A** Simulated behaviour of 10 (randomly chosen) individuals aged 6–18 years during a work day. Lines of different colours show the location of the 10 simulated individuals at every time of the day. **B** as A, but choosing individuals aged 19-64 years. **C** as A, but choosing individuals aged 65+ years.

School and workplace size are determined by sampling from the distribution of school and workplace size, as reported by the Education, Audiovisual and Culture Executive Agency of the European Commission and by the Italian National Institute of Statistics. The accuracy of the model in reproducing the descriptive statistics about employment, and school and workplace size is reported in [3].

1.2 Dynamic allocation of individuals in different settings

In the proposed model, each individual of the population is dynamically associated to a set of settings that includes its own household, its own school or workplace (if any) and the general community, which is assumed common to all individuals. At each time step of the simulation (representing a time interval of 10 minutes), each individual i is associated to a specific location and can have contacts only with individuals who are in the same location during the same time slot. The dynamic clustering of individuals over time is modelled according to the observed time-use data for the Italian population, collected over the period 2002-2003 [4]. In particular, time-use data were used to compute the probability of spending time at home, school, work or in the general community for individuals of different age and employment type (e.g. students vs workers) at different times of the day.

More in detail, let N be the overall number of simulated unemployed individuals of age between 61 and 80 years. We indicate with $N_H(t)$ the number of simulated unemployed individuals of age between 61 and 80 that are at home at time t and with $N_C(t)$ the number of simulated unemployed individuals of age between 61 and 80 that are in the general

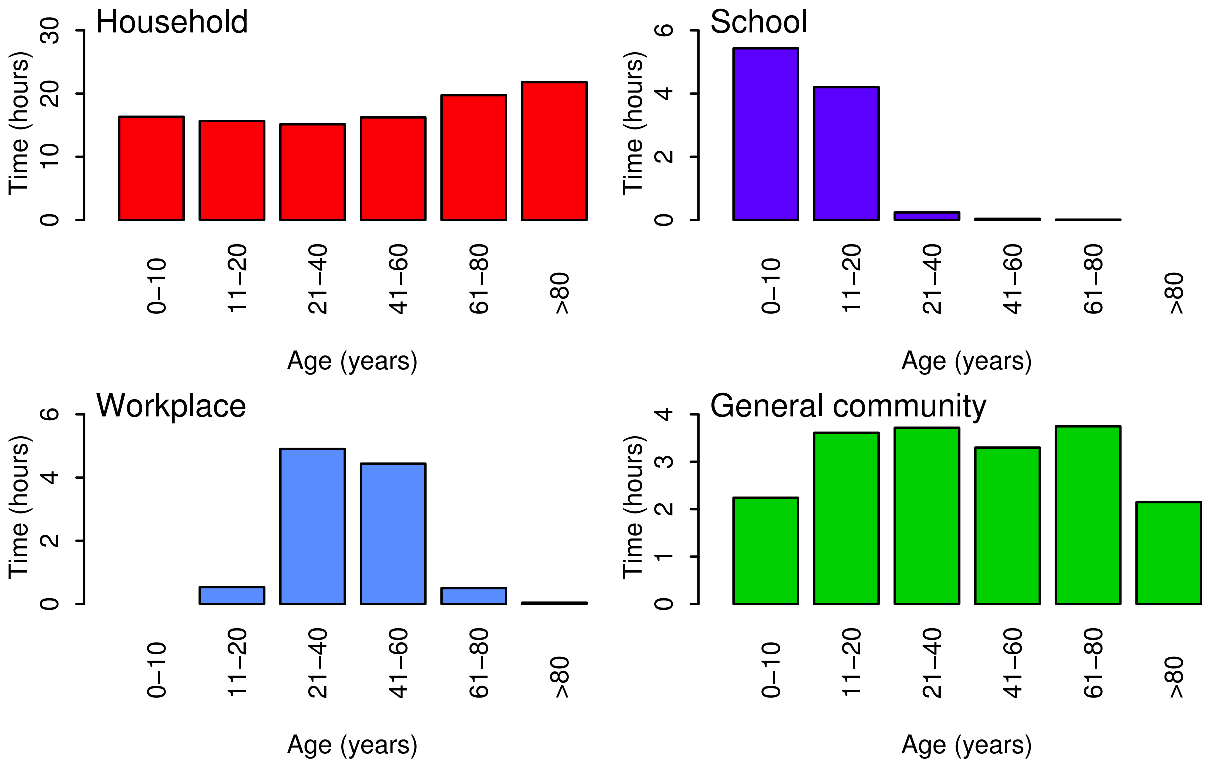

Figure S3: Cumulative time spent in the four modelled social settings during work days (Monday-Friday) for different age groups.

community at time t. Finally, let $p_H(t)$ and $p_C(t)$ be the fractions (with $p_H(t)+p_C(t)=1$) of individuals of age between 61 and 80 respectively at home and in the general community at time t according to the time-use survey.

Let us now consider a simulated unemployed individual i of age between 61 and 80. Individual i may switch between two settings, namely household and general community.

At the beginning of the simulation (midnight of the first simulated day), the probability distribution of being at home or in the general community is $B(p_H(0))$ where B is the Bernoulli distribution. Basically, we generate a random number from $B(p_H(0))$. If we get a success individual i is located at home, otherwise individual i is located in the general community. It follows that the average number of simulated individuals of age between 61 and 80 at home at time $t=0$ is $N_H(0)=Np_H(0)$ and thus the resulting fraction is $p_H(0)$. Similarly, the fraction of individuals of age between 61 and 80 in the general community at time $t=0$ is $p_C(0)$.

To determine the location at time $t=1$ we adopt the following algorithm:

- if individual i is located at home at time $t=0$
  - if $p_H(1) \geq p_H(0)$ individual i remains at home
  - if $p_H(1) \leq p_H(0)$ individual i is located in the general community with probability $1-p_H(1)/p_H(0)$; the probability distribution of moving to the general

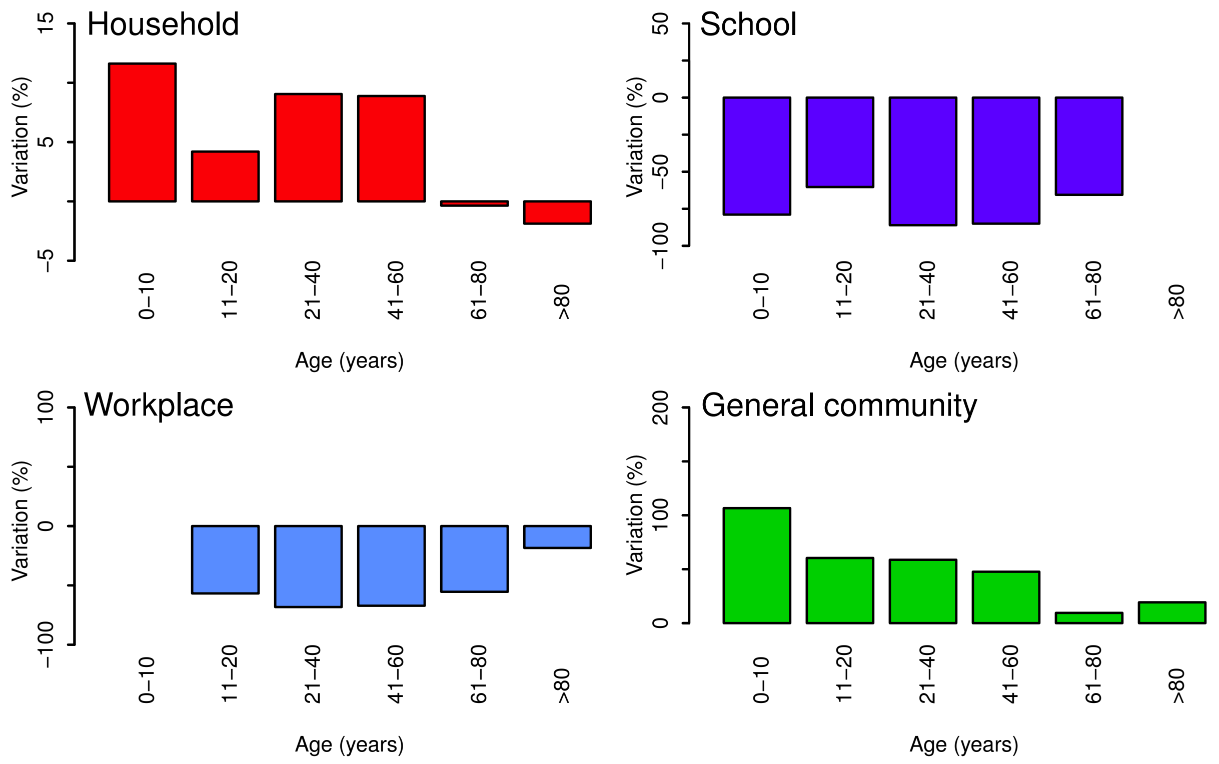

Figure S4: Percentage variation of cumulative time spent in the four modeled social settings during weekends (Saturday and Sunday) with respect to that spent in working days (Monday-Friday) for different age groups.

community or remaining at home is $B(1 - p_H(1)/p_H(0))$.

- if individual i is located in the general community at time $t = 0$
  - if $p_R(1) \geq p_R(0)$ individual i remains in the general community,
  - if $p_R(1) \leq p_R(0)$ individual i is located at home with probability $1-p_R(1)/p_R(0)$; the probability distribution of going home or remaining in the general community is $B(1 - p_R(1)/p_R(0))$.

It follows that if $p_H(1) \geq p_H(0)$ (and thus $p_R(1) \leq p_R(0)$), the average number of simulated individuals of age between 61 and 80 at home at time $t = 1$ will be

$$N_H(1) = N_H(0) + N_R(0)(1 - p_R(1)/p_R(0)) = N - N_R(0)p_R(1)/p_R(0).$$

Consequently, the fraction of simulated individuals of age between 61 and 80 at home at time $t = 1$ will be $1 - p_R(1) = p_H(1)$.

Analogously, if $p_H(1) \leq p_H(0)$ it can be seen that

$$N_H(1) = N_H(0) - N_H(0)(1 - p_H(1)/p_H(0))$$

and the fraction of simulated individuals of age between 61 and 80 at home at time $t = 1$ will be $p_H(1)$.

The procedure is iterated for all $t \geq 1$ and this simple calculation shows that the algorithm preserves fractions $p_H(t)$ and $p_R(t)$.

We used the above described procedure to dynamically locate in the different settings all unemployed individuals. We considered the following age groups of unemployed individuals: 0-10, 11-20, 21-40, 41-60, 61-80, ≥ 81.

A similar procedure was used to dynamically locate students and workers in the different settings by considering that in this case 3 settings are involved in the computation (household, general community, school/workplace). As for students, we considered the following age groups: 6-10, 11-13, 14-18, ≥ 19 in such a way to distinguish between students attending primary, lower and upper secondary schools, and universities.

This simple heuristic has the advantage to preserve the average daily time spent by individuals of different ages in the different settings (see Figure S1), avoiding unrealistic turnovers of individuals. The procedure could be improved (e.g. in our model, individuals are nor allowed to leave a setting if the probability of being in that setting, as resulting from time-use data, is increasing) but this would require computing complicated conditional probabilities, e.g. for conditioning the probability of leaving a certain setting to the time currently spent in that setting. However, Fig. S2 shows that the use of the heuristic model produces reasonable individual behaviours during the course of a day for individuals of different age.

Model simulations account for the difference between work days and weekends in such a way to follow a cyclic pattern composed by five simulated days where the probabilities are derived from time-use data referring to work days (Monday-Friday), followed by two simulated days where probabilities are derived from weekend days (Saturday and Sunday). Figure S3 shows the average time spent in different setting for different age groups during work days. Figure S4 shows, for different age groups, the variation of total amount of time spent in each setting during a weekend day with respect to a work day; for instance, individuals aged 11-20 years spend about 50% more time in the general community during weekend days with respect to work days.

1.3 Calibration of the disease transmission model

Influenza transmission is modeled by following the classic susceptible-latent-infectious-removed (SLIR) scheme. Susceptible individuals can get infected through contact with infectious individuals who are sharing the same place at the same time. The mixing between individuals sharing the same location at the same time is assumed to be homogeneous.

We assume the length of the latent period to be 1.5 days and the length of the infectious period to be 1.2 days. The resulting length of the generation time (i.e., the duration between the time of infection of a secondary case and the time of infection of its primary infector) is 2.7 days [2, 5, 6]. Recovered individuals are assumed to be fully protected. Initially immune individuals are randomly determined on the basis of age-specific seropositive rates as observed before the 2009 H1N1 pandemic in Italy [7, 8]. Simulations are initialized with 10 infected individuals and we do not consider importation of cases during

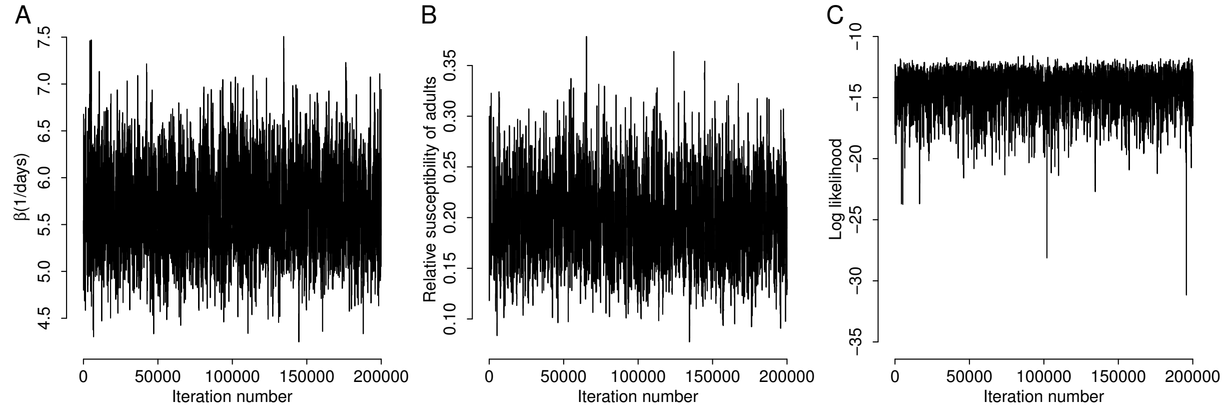

Figure S5: MCMC output at each iteration for model HSWR. **A** Transmission rate β. **B** Relative susceptibility of adults (19+ year-old) with respect to younger individuals $\bar{\rho}$. **C** Log-likelihood.

the course of the epidemic. Such a simplifying hypothesis can be considered a reasonable approximation since, despite the importation of cases has a strong impact in determining the timing and spatial spread of an influenza pandemic, it does affect neither the final attack rate by age nor the proportion of cases by setting, which are the quantities of major interest in this work. Another characteristics that has clearly arisen from the analysis of the data on the 2009 H1N1 pandemic is a pattern of differential susceptibility by age (see for instance [2, 7, 9, 10]). As in [2, 10], susceptibility to infection by age is modeled by dividing the population in two age groups: individuals aged 0-18, who are assumed to be fully susceptible to infection (to avoid over-parametrization) and adults (19+ years-old individuals).

We used MCMC for estimation of the two free parameters of models HR, HSR and HSWR, namely the transmission rate β and the relative susceptibility of adults $\bar{\rho}$, specifically random-walk Metropolis sampling in the logarithmic scale. The chain was initialized with β drawn form a uniform distribution $U[0, 15]$ and $\bar{\rho}$ drawn form a uniform distribution $U[0, 1]$. For model HSWR, at each iteration, if the current value of the transmission rate is β, a new value $\beta^\star = \beta \exp\{\delta u\}$ is generated, where $\delta = 0.2$ (for good mixing) and u is drawn from a normal distribution $\mathcal{N}(0, 1)$. The above described procedure was used because classical Metropolis sampling with Gaussian jumping distribution is not suitable when dealing with positive definite parameters - trivially, the transmission $\beta^\star$ could assume negative values by sampling from a Gaussian distribution. However, the adopted procedure is equivalent to sampling the logarithm of the transmission rate from a gaussian distribution, in fact $\log \beta^\star \sim \mathcal{N}(\log \beta, \delta^2)$ (see for instance [11, 12]).

We performed 200,000 iterations. The first 5,000 were discarded as burn-in period. We checked convergence by considering several different starting points and by visual inspection (see for instance Fig. S5). To get a set of independent samples – thus avoiding autocorrelation between adjacent samples – we considered one sample every 25 iterations. A similar procedure (with $\delta = 0.2$) was used to update $\bar{\rho}$. We used the same procedure for

Age group (years)	Seropositive (%)	
	Pre-pandemic	Post-pandemic
$0-5$	-	40.5 (95%CI: 34.9-46.4)
$6-18$	5.8 (95%CI: 2.1-12.1)	62.3 (95%CI: 53.7-70.4)
$19-64$	2.6 (95%CI: 1.4-4.3)	12 (95%CI: 9.1-15.4)
65+	15.7 (95%CI: 12.7-19.1)	17.3 (95%CI: 13.5-21.5)

Table S1: Percentage of seropositive individuals (HI titre $\geq 1:40$) by age groups as observed in the data collected before and after the 2009 H1N1 pandemic. 95% CI are calculated by using exact binomial test. Data adapted from Merler et al. [7].

calibrating models HR and HSR but with slightly different values of δ.

1.4 Serological data

The level of immunity against H1N1pdm in pre and post pandemic sera is taken from [7] and summarized in Tab. S1. Specifically, in that study the antibody titres were measured by the haemagglutination inhibition (HI) assay. Here we assume individuals to be seropositive when HI titre ≥ 40. In model simulations, the initial fraction of immune population in the four age groups considered in this study is assumed to be equal to the fraction of pre-pandemic seropositives as reported in Tab. S1.

1.5 Computation of reproduction numbers

An explicit equation to compute the basic reproduction number (R_0, representing the average number of infections generated by a typical infected individuals in a fully susceptible population) for individual based models is not available. Therefore, as already proposed in the literature (see for instance [2, 5, 13, 14]), we infer R_0 from the simulated epidemics. Specifically, by starting from the estimate of the epidemic growth rate and assuming a fixed (known) generation time, it is possible to estimate the basic reproduction number as

$$R_0 = (1 + rT_l)(1 + rT_i)$$

where T_l is the average length of the latent period, T_i is the average length of the infectious period, and r is the exponential rate of the epidemic. The exponential growth rate r can be estimated by fitting a linear model to the logarithm of the incidence over time.

As described in [15], we apply the same technique to estimate the effective reproduction number (R_e), which is a measure of the transmission potential of a disease spreading in a partailly immune population, as it has been the case of the 2009 H1N1 pandemic.

1.6 Computation of R^{index} and R_e^{index}

Another way to measure the transmission potential of a disease is R^{index}, which is defined as the average number of individuals infected by the first infectious individual (the index

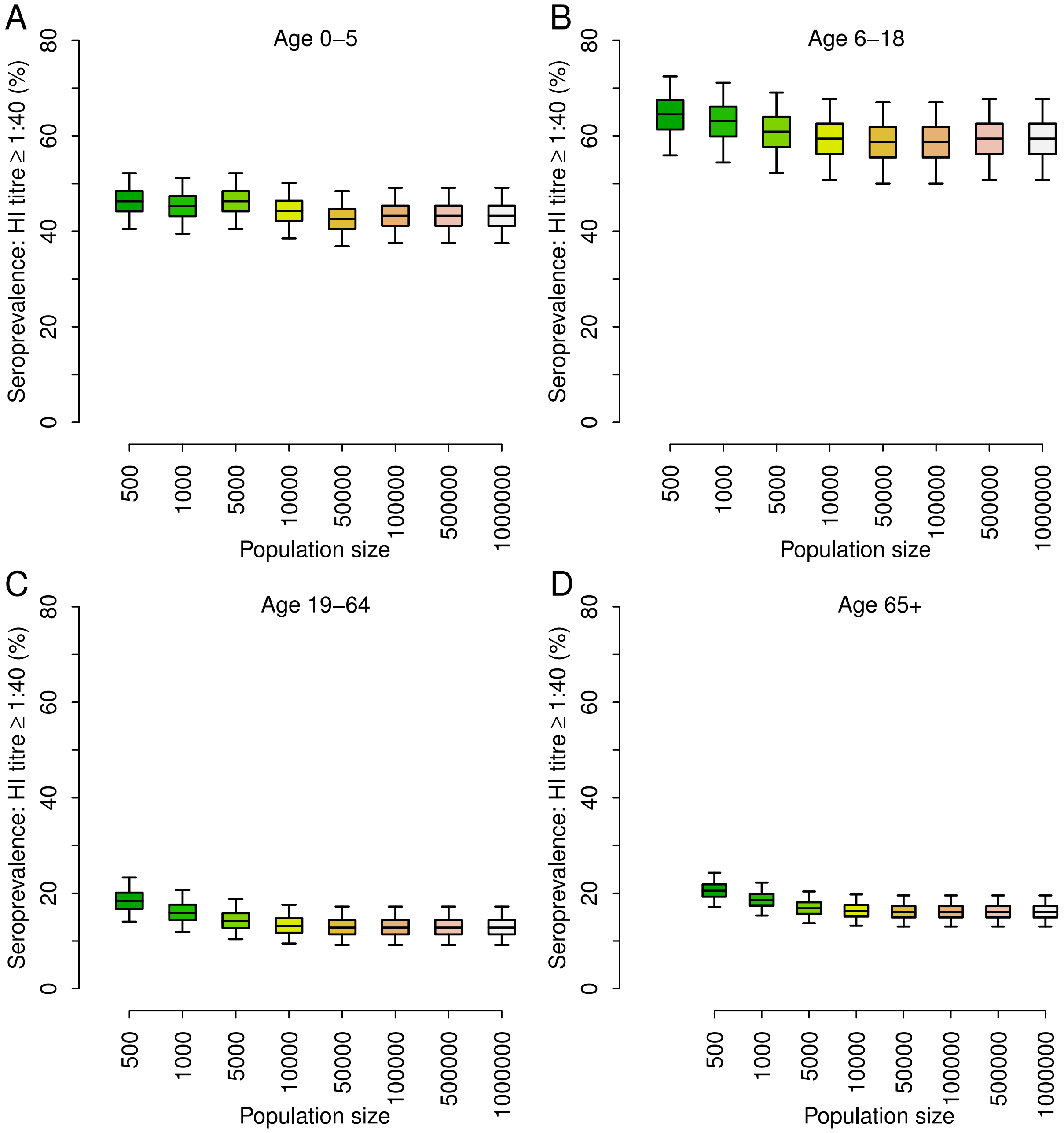

Figure S6: Posterior distribution (mean, 50%CI and 95%CI) of influenza seroprevalence by age group estimated with transmission model HSWR (calibrated by MCMC sampling as in the main text) for different values of the simulated population size.

case) in a fully susceptible population [6, 16]. The effective R^{index}, here denoted as R_e^{index}, is used as measure of the transmission potential when the population is not fully susceptible to the infection. This is appropriate in the case of 2009 H1N1 pandemic, as a fraction of the population is assumed to be fully immune to the infection at the beginning of the pandemic, according to age-specifc seroprevalence rates as measured before the start of the

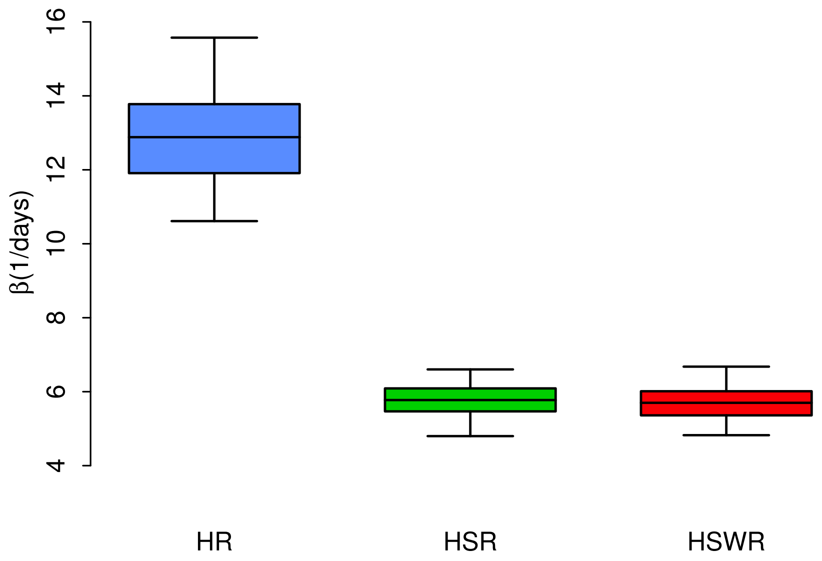

Figure S7: Posterior distribution (mean, 50%CI and 95%CI) of the transmission rate β (expressed in days^{-1}) as estimated by MCMC sampling for the three proposed models described in the main text.

2009 pandemic [8, 7].

The procedure for computing R_e^{index} is the following:

- let $(\beta_i, \bar{\rho}_i)$ be the transmission rate and the relative susceptibility to infection at the i-th step of the MCMC calibration procedure;
- let $R_e^{index}(\beta_i, \bar{\rho}_i)$ be the resulting mean of the number of secondary infections generated by a randomly chosen (non-immune) index case in the population. $R_e^{index}(\beta_i, \bar{\rho}_i)$ is estimated by averaging over 100 individual estimates of $R_e^{index}(\beta_i, \bar{\rho}_i)$ obtained through the following procedure:
  - randomly chose an individual among all non-immune individuals in the population (he/she will be the index case);
  - simulate the epidemic with parameters $(\beta_i, \bar{\rho}_i)$ initialized with one index case.
  - compute the individual estimate of $R_e^{index}(\beta_i, \bar{\rho}_i)$ as the number infections generated by the index case;
- R_e^{index} is estimated by averaging $R_e^{index}(\beta_i, \bar{\rho}_i)$ over all the instances of model parameters as resulting from the MCMC calibration procedure (burn-in period excluded, and considering one parameter set every 25 iterations - see Sec. 1.3).

The above described procedure is used to obtain estimates of R_e^{index} stratified by the age of the index case and the setting where (secondary) infections are generated.

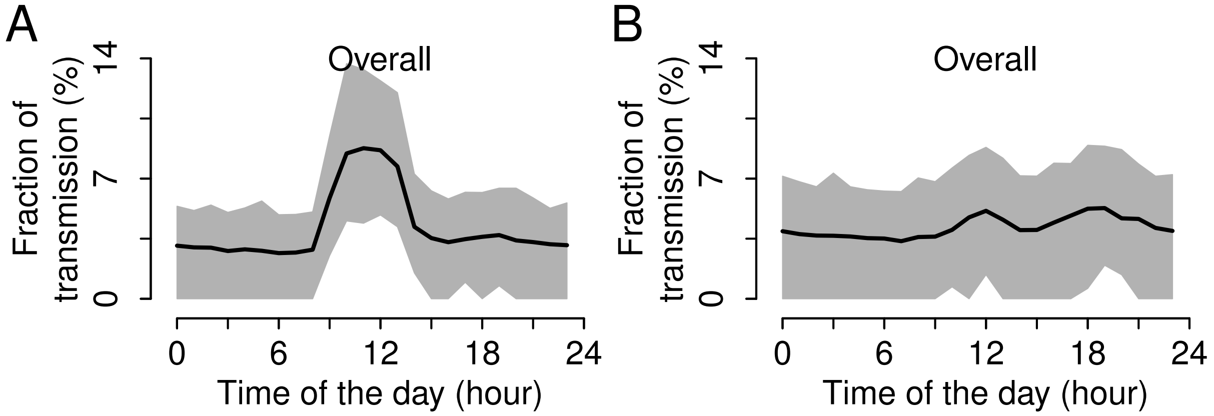

Figure S8: **A** Hourly percentage (average, lines, and 95%CI,colored areas) of daily transmission in a work day as estimated by model HSWR. Results refer to day 48 in a population of 100,000 individuals. **B** As **A** but for weekends.

2 Additional results

2.1 Robustness of results

The model was parametrized by exploring by Monte Carlo sampling the likelihood of the observed probability of being seropositive (HI titre ≥ 40) per age group at the end of the epidemic. The post-pandemic seroprevalence per age group as estimated by the different considered models is very stable with respect to the intrinsic stochasticity of the model. Moreover, as shown in Fig. S6, estimates are stable also with respect to changes in the size of the simulated population of individuals (for population size $\geq 10,000$ individuals). Therefore, in order to guarantee both the required robustness of the results and a reasonable computation time, all results presented in this manuscript are based on simulations performed on a population of 100,000 individuals.

2.2 Estimated influenza transmission rate

Fig. S7 shows the estimated distribution of the transmission rate β. The estimated distribution of β for model HSR (5.8 days^{-1}, 95%CI: 4.8-6.6) is very similar to that for model HSWR (5.7 days^{-1}, 95%CI: 4.9-6.7). The distribution of β for model HR is statistically different from that of models HSR and HSWR (12.9 days^{-1}, 95%CI: 10.7-15.6). Such a large average value of the transmission rate of model HR, together with the low estimated value of susceptibility to infection of adults with respect to children (see main text), is ascribable to the lack of schools contacts in model HR.

2.3 Within-day transmission dynamics

As highlighted in Fig. S8A, influenza transmission is highly variable during the course of a work day. In particular, a large fraction of daily cases occurs in the morning because

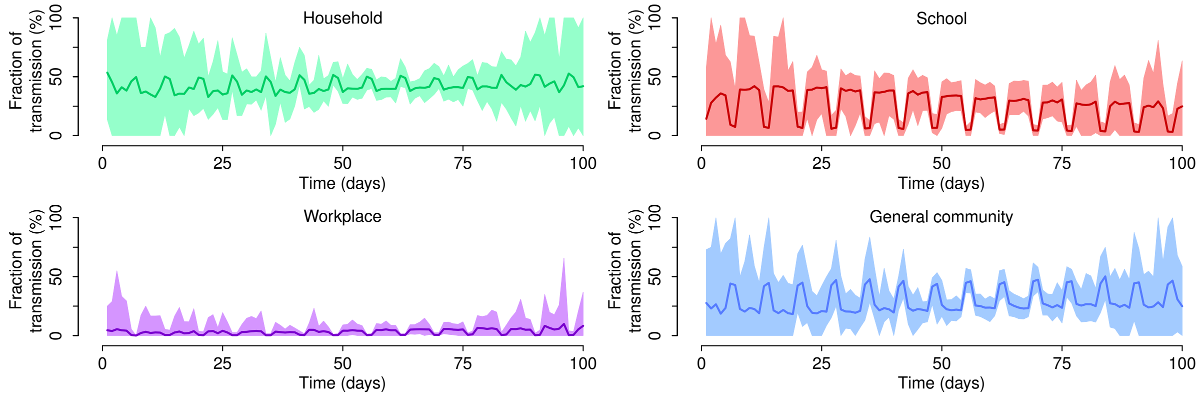

Figure S9: Daily percentage (and 95% CI, shaded areas) over time of infections in different social contexts as estimated by model HSWR in a population of 100,000 individuals.

of high rates of transmission in schools and, to a lesser extent, because of transmission in workplaces. A second small increase in disease transmission occurs around 6pm, mainly related to an increasing activity of individuals in the general community.

A different pattern of infection is predicted to occur during weekends, where the overall transmission do not show any marked increase during the course of the day. As shown in Fig. S8B, only two small increases in the transmission are predicted: the first one around midday, which is mainly ascribable to contacts in the general community and at school – in Italy the majority of schools are open on Saturday – and a second one around 7pm, mainly ascribable to contacts occurring in the general community.

Both in work days and weekends, a remarkable fraction of transmission occurs during the night, mainly determined by contacts between household members.

2.4 Influenza transmission by setting

Figure S9 shows the estimated transmission dynamics by setting during the course of an epidemic. Simulations show a high variability of estimates over time, especially in the initial phase of the epidemic and clearly highlight differences between work days and weekends. The period of high variability could be even much longer in larger populations (e.g. at country level). Moreover, a slightly larger fraction of transmission in schools is predicted in the early phase of the epidemic (while fraction of cases in other settings does not change much over time), supporting the idea that children tend to get infected earlier in the course of an influenza epidemic than adults. Such a pattern is also confirmed by the ILI data reported to the Italian surveillance system during the pandemic (see for instance [7]).

2.5 The role of weekend

To assess the role of weekends we assume weeks consisting of seven work days but the same model parameters (namely the transmission rate β and the relative susceptibility of adults $\bar{\rho}$) as estimated for models with weekends. Figure S10A and B shows, respectively, the

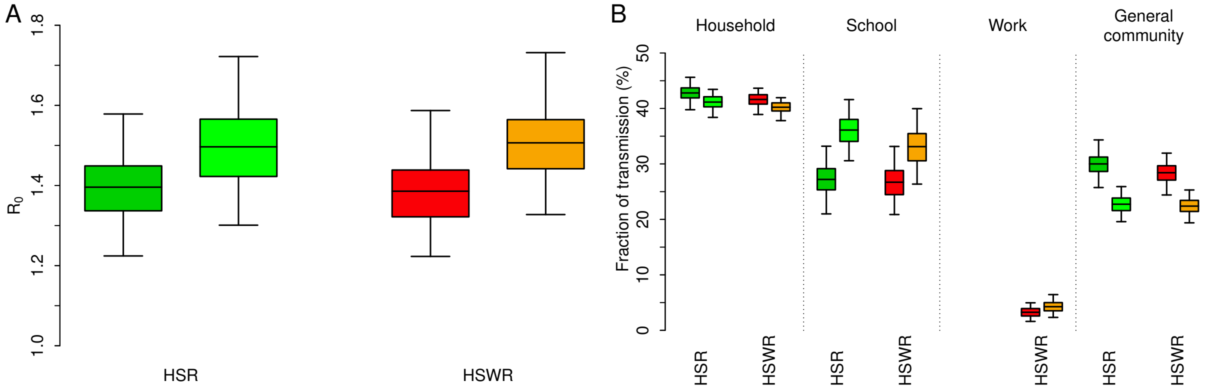

Figure S10: **A** Posterior distribution (mean, 50%CI and 95%CI) of the reproduction number estimated with transmission models HSR and HSWR. Dark colors refer to simulations where individuals follow a weekly schedule composed by five work days followed by two days of weekend; light colors refer to simulations where individuals follow a weekly schedule composed by work days only. **B** Posterior distribution (mean, 50%CI and 95%CI) of the proportion of influenza transmission in households, school, workplace and in the general community estimated with transmission models HSR and HSWR. Dark colors refer to simulations where individuals follow a weekly schedule composed by five work days followed by two days of weekend; light colors refer to simulations where individuals follow a weekly schedule composed by work days only.

estimated values of the reproduction number and the estimated fraction of transmission by setting, when weekends are considered or not. Results show that weekends (with more time spent in the general community and much less at school and work) are responsible for a reduction of R_0 of 6.7% on average according to model HSR (without weekends R_0 increases to 1.5, 95% CI 1.31-1.72), and a 8% reduction on average according to model HSWR (without weekends R_0 increases to 1.51, 95%1.33-1.73). We also found that the fraction of cases in different settings is affected by weekends. According to model HSWR, weekends are responsible for an increase in transmission in households of 3.5% on average (without weekends the fraction of cases in households decreases to 40.2%, 95% CI 37.9 - 41.9), a decrease in transmission in schools of 19.3% on average (without weekends the fraction of cases in schools increases to 33.1%, 95% CI 26.4-40), a decrease in transmission in workplaces of 23.3% (without weekends the fraction of cases in workplaces increases to 4.3%, 95% CI 2.4-6.4), and also for an increase in transmission in the general community of 26.8% on average (without weekends the fraction of cases in the general community decreases to 22.4%, 95% CI 19.5-25.3). According to model HSR, we found that without weekends the fraction of cases in households decreases to 41.2% (95% CI 38.7 - 43.4), the fraction of cases in schools increases to 36.1% (95% CI 30.6-41.6) and the fraction of cases in the general community decreases to 22.7% (95% CI 19.7-25.9).

References

[1] Merler S, Ajelli M (2010) The role of population heterogeneity and human mobility in the spread of pandemic influenza. Proc R Soc B 277: 557–565.

[2] Merler S, Ajelli M, Pugliese A, Ferguson NM (2011) Determinants of the Spatiotemporal Dynamics of the 2009 H1N1 Pandemic in Europe: Implications for Real-Time Modelling. PLOS Comput Biol 7: e1002205.

[3] Fumanelli L, Ajelli M, Manfredi P, Vespignani A, Merler S (2012) Inferring the Structure of Social Contacts from Demographic Data in the Analysis of Infectious Diseases Spread. PLOS Comput Biol 8: e1002673.

[4] ISTAT (2003). Indagine multiscopo sulle famiglie. Uso del tempo 2002-2003. (In Italian).

[5] Ferguson NM, Cummings DAT, Cauchemez S, Fraser C, Riley S, et al. (2005) Strategies for containing an emerging influenza pandemic in Southeast Asia. Nature 437: 209–214.

[6] Ferguson NM, Cummings DAT, Fraser C, Cajka JC, Cooley PC, et al. (2006) Strategies for mitigating an influenza pandemic. Nature 442: 448–452.

[7] Merler S, Ajelli M, Camilloni B, Puzelli S, Bella A, et al. (2013) Pandemic Influenza A/H1N1pdm in Italy: Age, Risk and Population Susceptibility. PLOS ONE 8: e74785.

[8] Rizzo C, Rota MC, Bella A, Alfonsi V, Declich S, et al. (2010) Cross-reactive antibody responses to the 2009 A/H1N1v influenza virus in the Italian population in the pre-pandemic period. Vaccine 28: 3558–3562.

[9] Cauchemez S, Donnelly CA, Reed C, Ghani AC, Fraser C, et al. (2009) Household transmission of 2009 pandemic influenza A (H1N1) virus in the United States. N Eng J Med 361: 2619–2627.

[10] Fraser C, Donnelly CA, Cauchemez S, Hanage WP, Van Kerkhove MD, et al. (2009) Pandemic Potential of a Strain of Influenza A (H1N1): Early Findings. Science 324: 1557–1561.

[11] Cauchemez S, Carrat F, Viboud C, Valleron AJ, Boëlle PY (2004) A Bayesian MCMC approach to study transmission of influenza: application to household longitudinal data. Stat Med 23: 3469–3487.

[12] Dorigatti I, Cauchemez S, Pugliese A, Ferguson NM (2012) A new approach to characterising infectious disease transmission dynamics from sentinel surveillance: Application to the Italian 2009–2010 A/H1N1 influenza pandemic. Epidemics 4: 9–21.

[13] Ajelli M, Merler S, Pugliese A, Rizzo C (2011) Model predictions and evaluation of possible control strategies for the 2009 A/H1N1v influenza pandemic in Italy. Epidemiol Infect 139: 68.

[14] Chowell G, Hengartner NW, Castillo-Chavez C, Fenimore PW, Hyman JM (2004) The basic reproductive number of Ebola and the effects of public health measures: the cases of Congo and Uganda. J Theor Biol 229: 119–126.

[15] Wallinga J, Lipsitch M (2007) How generation intervals shape the relationship between growth rates and reproductive numbers. Proc R Soc B 274: 599–604.

[16] Ajelli M, Merler S (2008) The impact of the unstructured contacts component in influenza pandemic modeling. PLOS ONE 3: e1519.
